# Systemic inflammation markers and cancer incidence in the UK Biobank

**DOI:** 10.1007/s10654-021-00752-6

**Published:** 2021-05-25

**Authors:** Therese Haugdahl Nøst, Karine Alcala, Ilona Urbarova, Karl Smith Byrne, Florence Guida, Torkjel Manning Sandanger, Mattias Johansson

**Affiliations:** 1 Department of Community Medicine, Faculty of Health Sciences, UiT The Arctic University of Norway, PO Box 6050, 9037 Langnes, Tromsø Norway; 2 Genomic Epidemiology Branch, International Agency for Research on Cancer, 150 Cours Albert Thomas, 69372 Lyon CEDEX 08, France

**Keywords:** Prospective cohort study, Cancer incidence, Blood-based inflammation markers, Systemic inflammation index, UK Biobank, Flexible parametric survival models

## Abstract

Systemic inflammation markers have been linked to increased cancer risk and mortality in a number of studies. However, few studies have estimated pre-diagnostic associations of systemic inflammation markers and cancer risk. Such markers could serve as biomarkers of cancer risk and aid in earlier identification of the disease. This study estimated associations between pre-diagnostic systemic inflammation markers and cancer risk in the prospective UK Biobank cohort of approximately 440,000 participants recruited between 2006 and 2010. We assessed associations between four immune-related markers based on blood cell counts: systemic immune-inflammation index (SII), neutrophil-to-lymphocyte ratio (NLR), platelet-to-lymphocyte ratio (PLR), lymphocyte-to-monocyte ratio (LMR), and risk for 17 cancer sites by estimating hazard ratios (HR) using flexible parametric survival models. We observed positive associations with risk for seven out of 17 cancers with SII, NLR, PLR, and negative associations with LMR. The strongest associations were observed for SII for colorectal and lung cancer risk, with associations increasing in magnitude for cases diagnosed within one year of recruitment. For instance, the HR for colorectal cancer per standard deviation increment in SII was estimated at 1.09 (95% CI 1.02–1.16) in blood drawn five years prior to diagnosis and 1.50 (95% CI 1.24–1.80) in blood drawn one month prior to diagnosis. We observed associations between systemic inflammation markers and risk for several cancers. The increase in risk the last year prior to diagnosis may reflect a systemic immune response to an already present, yet clinically undetected cancer. Blood cell ratios could serve as biomarkers of cancer incidence risk with potential for early identification of disease in the last year prior to clinical diagnosis.

**Supplementary Information:**

The online version contains supplementary material available at 10.1007/s10654-021-00752-6.

## Background

Inflammation may contribute to the incidence, tumour stage, and progression of cancer [1–3]. In diagnosed cancer patients, tumor-infiltrating lymphocytes serve as important clinical biomarkers for patient stratification and may complement traditional prognostic indicators, such as stage and grade [4–8]. Indeed, both the local increase in immune cell infiltration in tumors, as well as elevated systemic inflammation responses, may be important indicators of cancer progression and prognosis [9–11]. Furthermore, low-grade chronic inflammation, characterized by a persistent increase of inflammatory cells and pro-inflammatory mediators, are often elevated prior to cancer diagnosis, and may be cancer promoting [12].

Systemic inflammation can be assessed using various biochemical or haematological markers routinely measured in common blood tests or as ratios derived from these measurements [13]. Specifically, four ratios have been highlighted by previous studies as related to morbidity and mortality, including neutrophil-to-lymphocyte ratio (NLR), platelet-to-lymphocyte ratio (PLR), lymphocyte-to-monocyte ratio (LMR) and the systemic immune-inflammation index (SII) based on peripheral lymphocyte, neutrophil, monocyte and platelet counts [11, 14]. Although these ratios have been observed related to cancer risk and mortality, the majority of these studies have investigated them as prognostic markers in newly diagnosed cancer patients and have found inconsistent results [11, 15]. Nonetheless, two recent studies have assessed such markers pre-diagnostically; one study reported associations between blood cell ratios and total incidence of multiple cancers [16] in a Dutch cohort, and the other between blood cell counts and lung cancer incidence using UK Biobank data [17].

A better understanding of associations of blood cell ratios with cancer incidence risk is necessary to assess their potential as biomarkers for earlier identification of the disease. We aimed to evaluate the longitudinal relation between four systemic inflammation markers (SII, NLR, PLR, LMR) and site-specific cancer risk in the years leading up to diagnosis using data from UK Biobank, based on about 440,000 study participants. In order to assess differences in risk across years prior to diagnosis, we used flexible parametric survival models when estimating hazard ratios (HRs) for 17 cancer sites.

## Methods

### The UK Biobank and study participants

The UK Biobank is a large prospective cohort study of 502,540 men and women in the United Kingdom aged between 37 and 69 years at recruitment, which took place between 2006 and 2010. In addition to questionnaires, several physical measurements and one blood sample was collected from the participants [18]. The outcome of interest in this study was incidence of the most common cancers (with at least 50 incident cases in the UK Biobank to ensure both enough statistical power for analyses and anonymity of cases). The cancers included were bladder, brain, breast, colorectal, endometrium, kidney, liver, lung, lymphoma, myeloma, oesophagus, oral cavity/pharynx, ovary, pancreas, prostate, stomach and thyroid. Cancers were classified according to the International Classification of Diseases, 10^th^ Revision; see Supplementary Table S1. Follow-up for cancer incidence was conducted up to Feb 8, 2017, through linkage to national cancer registries (http://biobank.ndph.ox.ac.uk/showcase/showcase/docs/CancerLinkage.pdf). There were 442,115 eligible participants with blood cell measurements in this study (see inclusion flowchart in Supplementary Figure S1). Among these, 28,094 participants were diagnosed with cancer after blood sampling (time of inclusion) and during follow-up, and cancer-free participants (n = 414,021) were considered those participants with no cancer diagnosis registered until the end of follow-up (Feb 8, 2017). Additional information regarding cancer diagnoses (stage, tumor size etc.) was not available. All participants provided written consent, and the study protocol was approved by the North West Multicenter Research Ethics Committee in the United Kingdom.

### Systemic inflammation measurements and derived ratios

Peripheral blood samples of the UK Biobank participants were analysed at the UK Biobank central laboratory within 24 h of blood draw using a Beckman Coulter LH750 Hematology Analyzer. Thirty-one parameters were reported by the apparatus, from which individual blood cell populations were extracted. Specifically, neutrophil, lymphocyte and monocyte counts were extracted as calculated values by the instrument using differential blood cell counts (operating range 0.00–900.00 × 10^9^ cells/L). Platelet counts were extracted directly as instrument measurements (operating range 0.00–5000 × 10^9^ cells/L).

Based on peripheral blood cell counts, four systemic inflammation markers were calculated; SII, NLR, PLR, and LMR. Calculations were as follows; SII = (neutrophils * platelets)/lymphocytes, NLR = neutrophils/lymphocytes, PLR = platelets/lymphocytes, and LMR = lymphocytes/monocytes. These four ratios were considered in our analyses due to their reported pre-diagnostic associations to cancer risk and prognostic significance in cancer patients [11, 14].

### Statistical analyses

We estimated differences for each of the blood cell counts and ratios by sex and categories of age at recruitment using linear regressions. Up to 4.3% of observations were outside 1.5 times the interquartile ranges for any of the ratios, but were kept in the analyses and blood cell ratios were log-transformed and standardized (log(ratio)-mean(log(ratio))/sd(log(ratio)) prior to risk analyses. The main risk analysis involved modeling the relative hazards for risk of cancer for each site separately using flexible parametric survival models (rstpm2 v1.5.0, implementation of stpm2 in R v3.6.1 [19]). These models allow for capturing time-variant relationships between hazard ratios and blood cell ratios. The models include natural splines in intervals between two successive knots and can be used when the non-proportionality assumption of Cox models is violated, as was the case for the majority of models in this study. Years from blood sampling to end of follow-up (diagnosis of cancer, death, or end of follow-up, whichever occurred first) was used as time-scale in models. We estimated hazard ratios (HRs) associated with risk of cancer diagnosis per standard deviation increment in each log-transformed blood cell ratio, and included interaction terms with three knots; two boundary knots at follow-up time corresponding to 10% and 90% of incident cases for each cancer site, respectively, and one internal knot at 3 years of follow-up.

All models were stratified by sex, and the models were adjusted for: (1) age at blood draw only; (2) age at blood draw, blood C-reactive protein (CRP) concentrations, body mass index (BMI) at recruitment, and educational attainment level (minimally adjusted models); and (3) site-specific further adjustments for other known risk factors (fully adjusted models, Supplementary Table S2). The latter mentioned models were estimated only for cancers with known risk factors measured in UK Biobank [20] that were also associated with at least two of the four blood cell ratios in minimally adjusted risk analyses. CRP was included as a covariate in the models to represent an acute-phase inflammation biomarker.

Mean imputation was performed for variables with greater than 25% missing under a ‘missing at random’ assumption (see Supplementary Table S3). Imputation for continuous smoking variables was stratified by categories of age, sex, and smoking status. Additionally, we assumed no response in reporting for family history of prostate, colorectal, breast and lung cancer as ‘no family history’.

We carried out sensitivity analyses to evaluate the stability of the flexible survival models. Specifically, we evaluated whether the risks estimated by the models were influenced by extreme observations, specifications of model knots or our chosen imputation strategy (the latter using multiple imputation by chained equations (MICE, R package mice, v3.12.0) with predictive mean matching in addition to min and max imputations). Additionally in minimally adjusted models for colorectal cancer, information on regular use of the anti-inflammatory drug aspirin (424 users among cases and 56,809 among controls) was included as an additional covariate.

## Results

### General characteristics of the study population in the UK Biobank

Among the 442,115 participants included in our analyses, the majority were women (53.6%) and those subsequently diagnosed with cancer were, on average, 4 years older at recruitment compared to participants cancer-free at latest follow-up (60 vs. 56, range: 37–73) (Table 1; for more details see Supplementary Table S3). Further, the majority of participants were residents in England (~ 11% were from Scotland and Wales) and had at least a secondary school level education. During the average follow-up period of 8 years for all participants, 28,094 participants (6.4%) were diagnosed with cancer with an average follow-up time of 3 years (Table 1). At least 50 incident cancers were identified for 17 cancer sites: prostate (n = 4324), breast (n = 4237), colorectal (n = 2401), lung (n = 1458), lymphoma (n = 910), endometrial (n = 650), kidney (n = 537), bladder (n = 497), ovary (n = 429), pancreas (n = 420), oesophageal (n = 393), oral cavity/pharynx (n = 381), brain/central nervous system (n = 334), myeloma (n = 311), stomach (n = 273), liver (n = 202), and thyroid (n = 181). The number of reported cancers were 4828, 5036, 5497, 5566, and 5177 in the first five consecutive years of follow-up.

**Table 1:** Baseline characteristics of UK Biobank participants included in this study

Variable		Controls (cancer-free)	Cases
Total participants N (%)		414,021 (93.6)	28,094^a^ (6.35)
Sex N (%)	Male	190,326 (46.0)	14,749 (52.5)
	Female	223,695 (54.0)	13,345 (47.5)
Region N (%)	England (without London)	308,885 (74.6)	21,293 (75.8)
	London	58,964 (14.2)	2842 (10.1)
	Scotland	28,916 (6.98)	2674 (9.52)
	Wales	17,256 (4.17)	1285 (4.57)
Education N (%)	Primary School	67,616 (16.3)	6224 (23.1)
	Technical School	48,079 (11.6)	3615 (12.9)
	Secondary School	157,618 (38.1)	9584 (33.5)
	University	135,823 (32.8)	8327 (29.2)
	Unknown	4885 (1.18)	344 (1.28)
Age at blood collection (in years)	Mean (SD)	56.5 (8.11)	60.8 (6.81)
	Min–max	37.4–73.7	40.2–70.9
Age at diagnosis (in years)	Mean (SD)		63.9 (6.93)
	Min–max		40.4–77.7
Follow-up time (in years)	Mean (SD)	7.86 (0.95)	3.09 (1.80)
	Min–max	0.01–9.71	0.00—7.75
Person-years		3,254,651	86,848
Systemic inflammation markers			
SII	Mean (SD)	596 (570)	619 (415)
	Min–max	0.04–212,493	1.81–21,461
NLR	mean (SD)	2.35 (2.98)	2.44 (1.42)
	Min–max	0.00–1101	0.02–92.5
PLR	Mean (SD)	144 (861)	142 (72.3)
	Min–max	1.90–344,000	1.70–5661
LMR	Mean (SD)	4.99 (27.0)	5.09 (25.8)
	Min–max	0.01–5401	0.02–1901

LMR: lymphocyte-to-monocyte ratio; NLR: neutrophil-to-lymphocyte ratio; PLR: platelet-to-lymphocyte ratio; SII: systemic immune-inflammation index

^a^This number includes all cancer sites for the UK Biobank participants, and comprises of a few additional sites to those included in this study. The total number of each cancer site is greater than the total number of participants with cancer, since some participants have been diagnosed with two different cancers on the same day

Baseline values for the evaluated blood cell ratios are reported in Supplementary Table S4. Among controls, mean SII, NLR, and LMR values were higher in women than in men (*P* < 3.7 × 10^–12^), whereas NLR was higher in men. Among cases, mean NLR values were higher in men (*P* = 9.6 × 10^–71^) and mean PLR and LMR values were higher in women (*P* < 2.7 × 10^–04^). Among women, SII and NLR values were lower or similar in older participants, both in controls and cases, whereas among men, SII and NLR values were higher in older participants and PLR and LMR values slightly lower or similar in older participants (Supplementary Table S4).

### Systemic inflammation markers and cancer risk

**Fig. 1:**
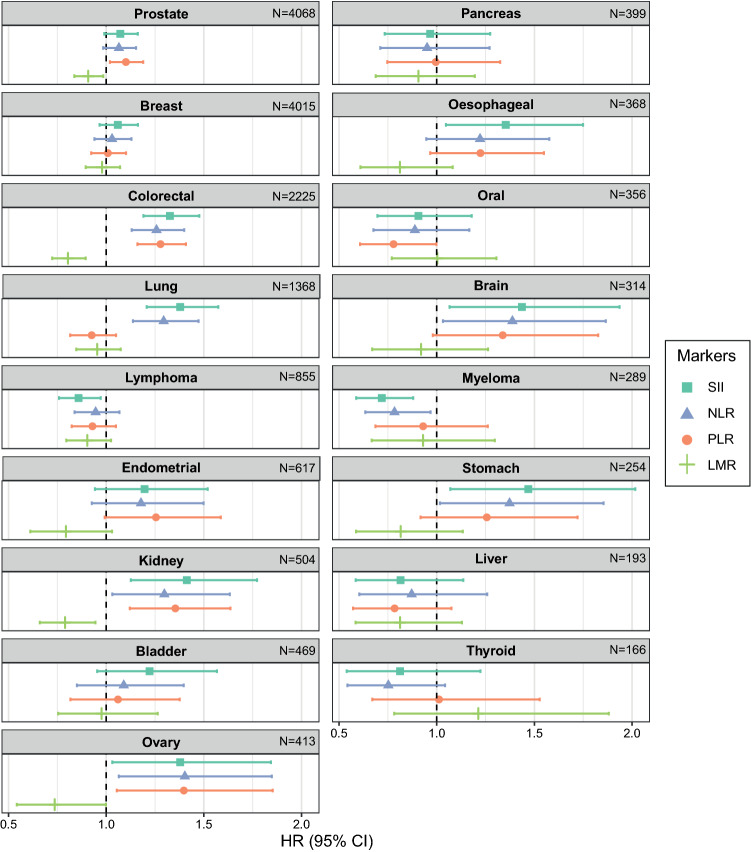
Overall hazard ratio (HR) estimates from minimally adjusted flexible survival models^a^ for all systemic inflammation markers and all cancers in this study. ^a^This figure present HR estimates from flexible parametric survival models that were stratified by sex and adjusted for age at blood draw, blood CRP concentrations, BMI and educational attainment level, and included an interaction with follow-up time. Abbreviations: BMI: Body mass index; CRP: C-reactive protein; HR: hazard ratio; LMR: lymphocyte-to-monocyte ratio; NLR: neutrophil-to-lymphocyte ratio; PLR: platelet-to-lymphocyte ratio; SII: systemic immune-inflammation index

In the initial risk analysis we found consistent positive associations of SII, NLR, and PLR, and negative associations for LMR with risk of colorectal, kidney, and ovarian cancer (Fig. 1 and Supplementary Table S5). We observed the strongest associations for these three cancers. We also observed associations for at least two inflammation markers with risk of prostate, lung, brain, myeloma, and stomach cancers (Fig. 1 and Supplementary Table S5). Additionally, we observed negative associations between SII and risk of lymphoma and between PLR and risk of oral cancer, and a positive association between SII and risk of oesophagal cancer. When adjusting for age at blood draw, blood CRP concentrations, BMI at recruitment, and educational level, overall risk estimates were qualitatively similar to those from crude models (Supplementary Table S5). Similar risk estimates were also observed when adjusting models for additional site-specific risk factors in fully adjusted models. In fully adjusted models, all four blood cell ratios were associated with risk of colorectal, kidney and ovarian cancer, but not other cancers (Supplementary Table S5).

**Fig. 2:**
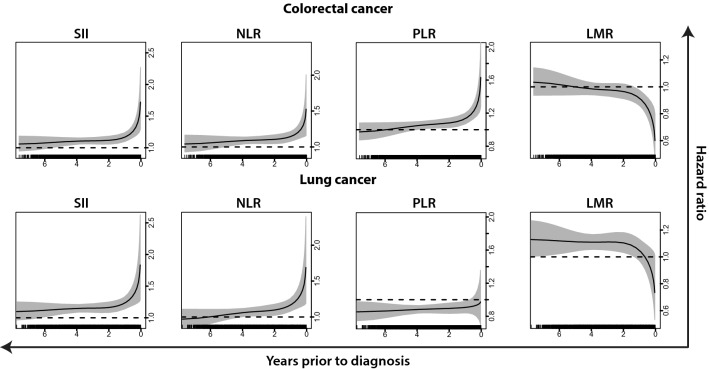
Figure depicts inflammation marker-specific HR estimates as a function of follow-up time for colorectal and lung cancer, as attained from the minimally adjusted flexible survival models.^1^ Rug plots depict the distribution of individual cancer cases over the follow-up period. ^1^This figure presents HR estimates as function of follow-up time from flexible parametric survival models that were stratified by sex and adjusted for age at blood draw, blood CRP concentrations, BMI and educational attainment level, and included an interaction with follow-up time. Abbreviations: BMI: Body mass index; CRP: C-reactive protein; HR: hazard ratio; LMR: lymphocyte-to-monocyte ratio; NLR: neutrophil-to-lymphocyte ratio; PLR: platelet-to-lymphocyte ratio; SII: systemic immune-inflammation index

For colorectal and lung cancer, the associations between systemic inflammation markers and risk varied by time between blood draw and diagnosis (*P* values for interaction with time at the boundary knot closest to diagnosis were 2.0 × 10^–3^ and 1.0 × 10^–2^, respectively). Moreover, no clear association with risk of these two cancers for any of the markers was observed until 4–5 years prior to diagnosis, with subsequent markedly elevated HR estimates within the last year before diagnosis (Fig. 2 and Supplementary Figure S2). For example, the HR for colorectal cancer per standard deviation increment in SII in the minimally adjusted model was estimated at 1.09 (95% CI 1.02–1.16) five years prior to diagnosis and 1.50 (95% CI 1.24–1.80) one month prior to diagnosis, with similar observations for NLR and PLR. As expected, LMR displayed an increasingly pronounced negative association with colorectal cancer risk as time between recruitment and diagnosis decreased, with HR 1.00 (95% CI 0.94–1.06) five years prior to diagnosis and 0.70 (95% CI 0.58–0.85) one month prior to diagnosis. The differences in risk estimates across follow-up times were comparable across colorectal and lung cancer for SII and NLR, but less similar for PLR and LMR. For kidney, prostate, endometrial, and ovarian cancer, risks were also higher within the last year before diagnosis, but the overall interaction of risk with time was not significant (results not presented).

Although overall HR estimates did not change considerably after adjustments, the associations of the blood cell ratios with risk of cancer closer to diagnosis were attenuated when adjusted for CRP concentrations, current BMI and educational level (see risk profile according to follow-up time for colorectal cancer and SII as an example in Supplementary Figure S3A). We observed that adjustment for CRP moderately attenuated the association with risk close to diagnosis; e.g. considering models for SII and colorectal cancer as examples, the HR estimate at one month was 1.70 (95% CI 1.46–1.98) in the unadjusted models, 1.52 (95% CI 1.26–1.82) when adjusting for CRP, 1.50 (95% CI 1.24–1.80) in the minimally adjusted models and 1.49 (95% CI 1.24–1.79) in the fully adjusted models.

### Sensitivity analyses

For survival models, there were small differences in time-varying risks and Akaike information criterion from the models with different internal knots (see models for colorectal cancer and SII as an example in Supplementary Figure S3B). Moreover, model estimates were also not considerably influenced by the chosen imputation strategy, where MICE, min and max imputation was performed as sensitivity analyses (Supplementary Figure S2). Additional sensitivity analyses, presented for models for colorectal cancer, involved evaluating the influence of participants with extreme CRP concentrations or lymphocyte counts or with regular aspirin use, but excluding such participants did not affect the HR estimates (Supplementary Table S5).

## Discussion

In this large prospective cohort study of systemic inflammation and cancer risk, we observed positive associations for SII, NLR, and PLR, and negative associations for LMR, for seven of the 17 studied cancer sites. These associations were strong and consistent for colorectal and lung cancer, and they became more pronounced in the last year leading up to diagnosis. For these two cancers, the increased risks could represent systemic inflammation responses triggered by early stages of a clinically undetected cancer. Further, these results indicate that blood cell ratios could serve as potential biomarkers for cancer incidence and earlier identification of disease. Amongst the inflammation markers evaluated, SII generally displayed the strongest association with cancer risk and was associated with six of the 17 sites evaluated (colorectal, kidney, liver, lung, lymphoma and myeloma cancers).

This is the first prospective study with sufficient sample size to evaluate the association of systemic inflammation markers with risks of common cancer sites. Our results for colorectal, kidney, and ovarian cancer are largely consistent with that of a previous prospective study from the Netherlands that reported increased risk of overall cancer with elevated SII [16], and suggestive positive associations for lung and colorectal cancer. However, that study also observed positive associations of SII with risk of prostate cancer (HR 1.74 (95% CI 1.17–2.58)) [16], whereas we did not observe an association with SII for prostate cancer in this study (HR 1.03 (95% CI 1.00–1.07)), and only weak associations to PLR and LMR. The difference in HR estimates between the two studies could be explained by fewer participants (n = 733 cancer cases among 8024 participants), older participants and more smokers in the Dutch study as compared to the UK Biobank participants. Of note, there were no associations of any of the blood cell ratios to breast cancer in our study, which is in agreement with the observations in the Dutch study [16]. The lack of associations between systemic inflammation markers and breast cancer suggest that systemic inflammation markers could be less informative for certain cancers, such as breast, compared with colorectal and lung cancers.

A recent study using UK Biobank data, but limited to lung cancer and blood cell counts, has observed elevated white blood cell counts, especially neutrophils, associated to increased cancer risk [17]. We did not investigate single blood cell populations and risk of cancer in our study, but those study results are in agreement with our observations of higher SII and NLR values in cancer cases, also for lung cancers, which indicates that the associations to ratios could likely be driven by higher neutrophil counts. The systemic immune-related markers considered in this study are based on measurements that are available in routine laboratory analyses in larger hospitals in many countries. Further studies should target such biomarkers to evaluate whether predictive abilities of markers derived from such readily available measurements could aid identification of cancer during early diagnostic assessments.

Whilst we observed clear associations between inflammation markers and overall risk of several cancers, an important observation was that the HR estimates became notably more pronounced in the last year leading up to diagnosis, with little evidence for risk associations in blood drawn more than 5 years before diagnosis. HR estimates were moderately attenuated especially in the last year before cancer diagnosis when adjusting for blood CRP concentrations, which have been reported as elevated during the development of cancer disease [21]. Thus, the observed associations could reflect systemic immune responses possibly related to early stages of the disease, which are not solely acute inflammations (as represented by CRP). Further, the most prominent associations were observed in the short period leading up to diagnosis, which suggests that the associations reflect systemic immune response to pre-clinical cancer, rather than a causal etiological predisposition. We note that these specific results were not consistent with that of the Dutch study [16], as they observed elevated risks of incident cancers up to 8 years prior to diagnosis. Whilst the Dutch study included a longer follow-up period, the results were based on about 8000 study participants (733 cancer cases) in comparison to our analysis in UK Biobank data, including over 440,000 study participants (28,094 cancer cases). It is not evident why we observed stronger associations in the last year before cancer diagnosis, whilst the Dutch study with a longer follow-up time observed stronger associations many years before diagnosis.

Indeed, the primary strength of our study is the large study sample and high number of cancer cases, which allowed for assessment of cancer-specific associations to systemic inflammation markers and careful modeling of their longitudinal relation with cancer risk. Clearly, the relation between systemic inflammation markers and subsequent cancer diagnosis varied by follow-up time for several cancers and did not support a causal relation with the predisposition of incident cancer. Rather than excluding cancer cases diagnosed within one year of blood sampling we chose an approach using non-linear modeling of follow-up time. Sensitivity analyses demonstrated robustness of our results to specifications of the flexible parametric survival models, imputations and extreme observations. We presented crude *P* values for the survival models, which could be influenced by Type 1 errors. However, assuming all tests were independent, which is likely not the case for the different ratios, the main results of this study would still be considered significant after Bonferroni adjustment (4 ratios * 17 cancer types * 4 models = 272 tests, *P* = 0.05/272 = 1.84e-04; models significant for colorectal and lung cancers in Table S5 have *P*-values < 8.64e-05).

We note that the current analysis cannot evaluate the extent to which advanced disease is driving the relations observed as this information is not available in the UK Biobank cohort. Further, the analysis was limited to 8 years of follow-up, but as UK Biobank participants continue to be followed and more incident cancers are identified, the cohort will allow for additional more detailed analyses by histological subtypes and other tumor characteristics in the future.

## Conclusions

We observed overall moderate associations of systemic inflammation markers with risk of several cancers. The strongest associations were found for colorectal and lung cancer, with particularly pronounced associations in the year leading up to diagnosis. The clear associations between inflammation markers and risk close to cancer diagnosis likely reflect the systemic response to a developing cancer and would not be consistent with causal predisposition of these immune characteristics. We conclude that systemic immune response is an important pre-clinical feature in the advanced development of colorectal and lung cancer, and blood cell ratios could serve as biomarkers of cancer incidence risk with potential for early identification of disease in the last year prior to clinical diagnosis.

## Supplementary Information

Below is the link to the electronic supplementary material.

Supplementary file1 (DOCX 812 KB)

## Data Availability

The UK Biobank is an open access resource, available at https://www.ukbiobank.ac.uk/researchers/.

## Code availability

The statistical analyses were performed using custom codes, which are available upon request.
