# Supplementary material for: Systemic inflammation markers and cancer incidence in the UK Biobank
Source: Eur J Epidemiol. 2021 May 25;36(8):841–8. doi: 10.1007/s10654-021-00752-6 (PMC8416852; doi:10.1007/s10654-021-00752-6)
Supplement: Supplementary file 1 — Supplementary file1 (DOCX 812 KB) [file 10654_2021_752_MOESM1_ESM.docx]

**Supplementary materials for the research article**

**Systemic inflammation markers and cancer incidence in the UK Biobank**

Therese Haugdahl Nøst^1†*^, Karine Alcala^2†^, Ilona Urbarova^1†^, Karl Smith Byrne^2^, Florence Guida^2^, Torkjel Manning Sandanger^1^, Mattias Johansson^2^.

^†^Joint ﬁrst authors.

^1^ Department of Community Medicine, Faculty of Health Sciences, UiT The Arctic University of Norway, Tromsø, Norway;

^2^ Genetic Epidemiology Group, International Agency for Research on Cancer, Lyon, France.

**Figure S1**. Flowchart of the study population inclusion.

502,540 participants in the UK Biobank study

Participants with prevalent cancer* (n=37,541)

464,999 participants

Participants withdrawn (n=31)

Participants with blood sample after diagnosis (n=4)

Participants without blood sample (n=22,048)

Participants with missing data in ratios (n=801)

442,115 participants included in the present analysis

* Participants diagnosed with cancer prior to the recruitment date into UK Biobank.

**Figure S2.** Fully adjusted models using different imputation strategies (Min, Mean, Max, MICE). Rug plots display the distribution of individual cancer cases along the x-axis. The confidence intervals overlap in figures and their combined color appears as green. The estimates from MICE imputation are presented for one imputed dataset for each cancer type and ratio, but were only marginally different in five dataset imputations performed.

**
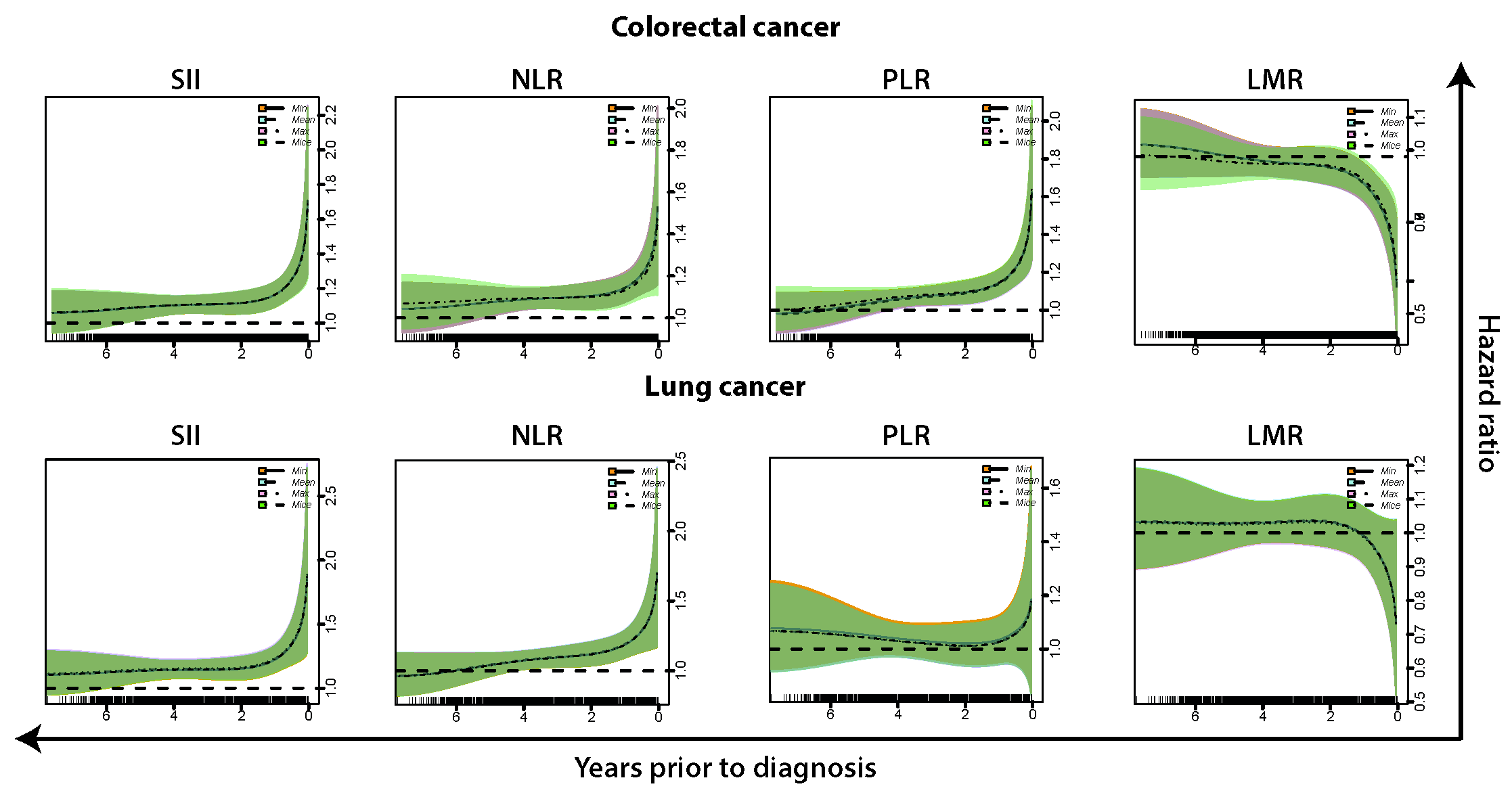
**

*Abbreviations: LMR: lymphocyte-to-monocyte ratio; NLR: neutrophil-to-lymphocyte ratio; PLR: platelet-to-lymphocyte ratio; SII: systemic immune-inflammation index.*

**Figure S3.** Hazard ratios according to follow-up time for colorectal cancer and systemic immune-inflammation index (SII). **A.** Crude and two adjusted models (adjustment with CRP only, and adjustment with C-reactive protein (CRP), body mass index (BMI) and educational level). **B.** Crude models with different placements of the internal knot. Boundary knots are placed at 10% and 90% of the cancer cases. The Akaike information criterion at 1, 2, 3, and 5 years and at median follow-up time was 38036, 38033, 38031, 38028, and 38031, respectively.


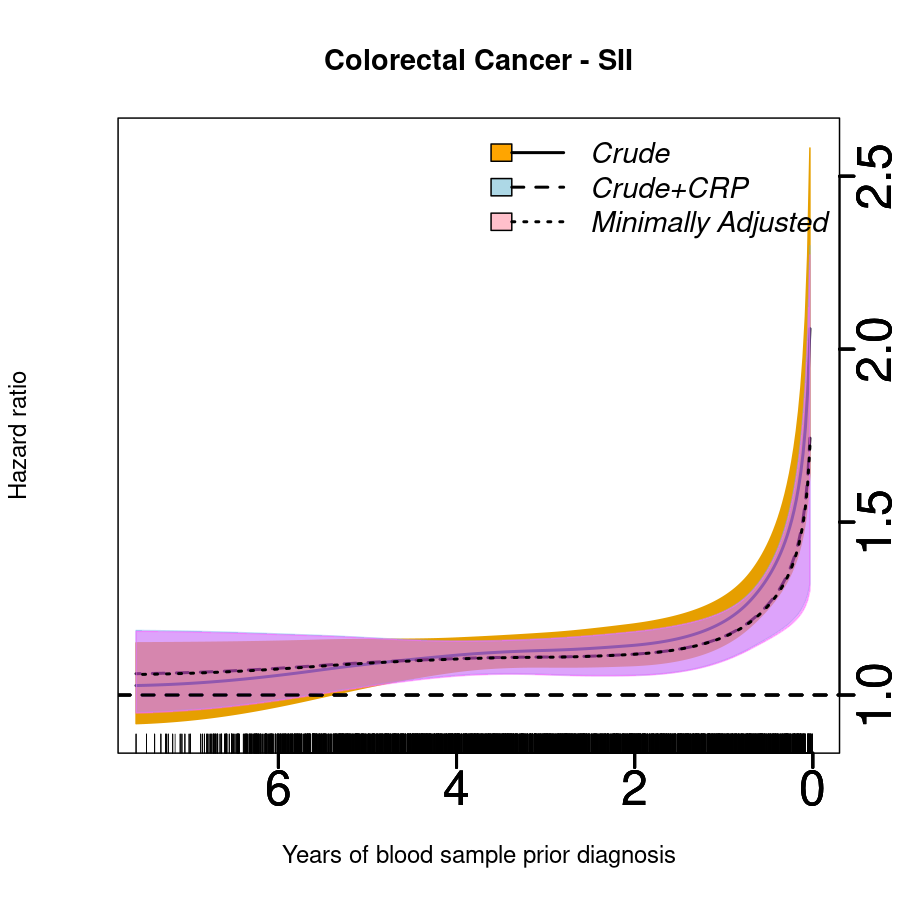

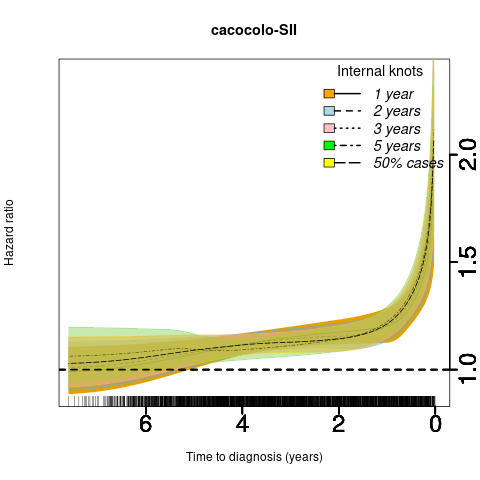


**Years prior to diagnosis**

**Hazard ratio**

**Years prior to diagnosis**

**Hazard ratio**

**A**

**B**

*Abbreviations: BMI: Body mass index; CRP: C-reactive protein; SII: systemic immune-inflammation index.*

**Table S1**. Cancer classification according to the International Classification of Diseases, 10^th^ Revision (ICD10).

| **Cancer site** | **Topographical code** |
| --- | --- |
| Bladder | C67 |
| Brain and CNS | C70-C72 |
| Breast | C50 |
| Colorectal | C18-C20 |
| Endometrium | C54-C55 |
| Kidney | C64 |
| Liver | C22 |
| Lung | C33-C34 |
| Lymphoma | C81-C86 |
| Myeloma | C90 |
| Oesophagus | C15 |
| Oral Cavity and Pharynx | C00-C06, C09,C10, C12-C14 |
| Ovary | C56 |
| Pancreas | C25 |
| Prostate | C61 |
| Stomach | C16 |
| Thyroid | C73 |

**Table S2**. Overview of variables included in the fully adjusted flexible survival models in this study.

| **Cancer site** | **Risk factors** | **Model specification notes** |
| --- | --- | --- |
| Prostate | Age, family history of prostate cancer, BMI, CRP, educational level |  |
| Colorectal | Age, family history of bowel cancer, waist to hip ratio, cigarette pack-years, frequency of processed meat intake (<1 per week vs. ≥1 per week), moderate physical activity (days per week), weekly alcohol intake (grams), BMI, CRP, educational level |  |
| Lung | Age, family history of lung cancer, cigarettes per day (0 for never smokers), years of smoking (0 for never smokers), smoking status (never vs former vs current), PM2.5 level in 2010 (microg/m^3^), BMI, CRP, educational level |  |
| Kidney | Age, BMI, smoking status (never vs. former vs. current), cigarette pack-years, ever diagnosed with hypertension, CRP, educational level |  |
| Ovary | Age, family history of breast cancer, parity (≥1 live birth vs. none), menopausal status (pr×10-menopausal vs. post-menopausal vs. unknown or hysterectomy), ever used hormone replacement therapy, duration of oral contraceptive use (never used (0) vs. <20 years vs. ≥20 years), BMI, CRP, educational level | Interactions:  (BMI)*(Menopausal status) |
| Stomach | Age, BMI, CRP, educational level |  |

*Abbreviations: BMI: Body mass index; CRP: C-reactive protein.*

**Table S3.** An extended descriptive characteristics of UK Biobank participants included in this study.

| **Variables** |  | **Controls (cancer-free)** | | **Cases** | |
| --- | --- | --- | --- | --- | --- |
|  |  | **N** | **%** | **N** | **%** |
|  |  | 414021 | 93.6 | 28094 ^1^ | 6.35 |
| **BMI (kg/m^2^)** | mean (SD) | 27.4 (4.79) |  | 27.6 (4.69) |  |
|  | min-max | 12.1-74.7 |  | 13.1-69.0 |  |
| **BMI (kg/m^2^)** | <18.5 | 2111 | 0.51 | 115 | 0.41 |
|  | [18.5-25] | 134866 | 32.6 | 8455 | 30.1 |
|  | [25-30] | 175249 | 42.3 | 12403 | 44.1 |
|  | [30-35] | 71894 | 17.4 | 5083 | 18.1 |
|  | [35-40] | 20339 | 4.92 | 1414 | 5.03 |
|  | >40 | 7897 | 1.91 | 519 | 1.85 |
|  | Unknown | 1665 | 0.4 | 105 | 0.37 |
| **Waist-to-hip ratio** | mean (SD) | 0.87 (0.09) |  | 0.89 (0.09) |  |
|  | min-max | 0.20-2.23 |  | 0.60-1.28 |  |
| **Smoking status** | Never smoker | 228345 | 55.2 | 13570 | 48.3 |
|  | Former smoker | 139602 | 33.7 | 11082 | 39.4 |
|  | Current smoker | 44024 | 10.6 | 3285 | 11.7 |
|  | Unknown | 2050 | 0.5 | 157 | 0.56 |
| **Smoking quantity (pack-years) ^2, 3^** | mean (SD) | 22.8 (4.79) |  | 27.6 (4.69) |  |
|  | min-max | 0.00-336 |  | 0.00-215 |  |
|  | Unknown | 60861 |  | 4386 |  |
| **Smoking quantity (cigarettes per day) ^2, 3^** | mean (SD) | 5.46 (10.2) |  | 7.26 (11.9) |  |
|  | min-max | 0.00-140 |  | 0.00-100 |  |
|  | Unknown | 91173 |  | 6839 |  |
| **Smoking duration (years) ^3^** | mean (SD) | 9.05 (14.4) |  | 12.4 (16.9) |  |
|  | min-max | 0.00-63.1 |  | 0.00-64.6 |  |
|  | Unknown | 61649 | 14.9 | 4255 | 15.1 |
| **PM 2.5 (micro-g/m^3^)** | mean (SD) | 10.0 (1.06) |  | 9.95 (1.07) |  |
|  | min-max | 8.17-21.3 |  | 8.17-18.5 |  |
|  | Unknown | 33523 | 8.1 | 2935 | 10.4 |
| **CRP (mg/L)** | mean (SD) | 2.54 (4.24) |  | 2.92 (4.72) |  |
|  | min-max | 0.08-80.0 |  | 0.08-80.0 |  |
|  | Unknown | 18688 | 4.51 | 1208 | 4.3 |
| **Father lung cancer ^3^** | Yes | 20850 | 5.04 | 1706 | 6.07 |
|  | No | 40030 | 9.67 | 3107 | 11.1 |
|  | Unknown | 353141 | 85.3 | 23281 | 82.9 |
| **Mother lung cancer ^3^** | Yes | 8883 | 2.15 | 668 | 2.38 |
|  | No | 30430 | 7.35 | 2412 | 8.59 |
|  | Unknown | 374708 | 90.5 | 25014 | 89 |
| **Sibling lung cancer ^3^** | Yes | 3201 | 0.77 | 313 | 1.11 |
|  | No | 17452 | 4.22 | 1725 | 6.14 |
|  | Unknown | 393368 | 95 | 26056 | 92.7 |
| **Father colorectal cancer ^3^** | Yes | 13090 | 3.16 | 1069 | 3.81 |
|  | No | 47790 | 11.5 | 3744 | 13.3 |
|  | Unknown | 353141 | 85.3 | 23281 | 85.9 |
| **Mother colorectal cancer ^3^** | Yes | 11704 | 2.83 | 962 | 3.42 |
|  | No | 27609 | 6.67 | 2118 | 7.54 |
|  | Unknown | 374708 | 90.5 | 25014 | 89 |
| **Sibling colorectal cancer ^3^** | Yes | 3993 | 0.96 | 379 | 1.35 |
|  | No | 16660 | 4.02 | 1659 | 5.91 |
|  | Unknown | 393368 | 95 | 26056 | 92.7 |
| **Father prostate cancer ^3^** | Yes | 26940 | 6.51 | 2038 | 7.25 |
|  | No | 33940 | 8.2 | 2775 | 9.88 |
|  | Unknown | 353141 | 85.3 | 23281 | 82.9 |
| **Sibling prostate cancer ^3^** | Yes | 4762 | 1.15 | 566 | 2.01 |
|  | No | 15891 | 3.84 | 1472 | 5.24 |
|  | Unknown | 393368 | 95 | 26056 | 92.7 |
| **Parity** | Yes (>1 live birth) | 181193 | 43.8 | 10951 | 39 |
|  | No (0 live birth) | 41963 | 10.1 | 2369 | 8.43 |
|  | Unknown | 190865 | 46.1 | 14774 | 52.6 |
| **Duration of oral contraception (women only) ^3^** | Never | 40857 | 18.3 | 2871 | 21.5 |
|  | 0-20 years | 136745 | 61.1 | 7880 | 59 |
|  | > 20 years | 24955 | 11.2 | 1225 | 9.18 |
|  | Unknown | 21138 | 9.45 | 1369 | 10.3 |
| **Menopausal status (women only)** | Pr×10-Menopause | 53115 | 23.7 | 1952 | 14.6 |
|  | Post-Menopause | 160970 | 72 | 10914 | 81.8 |
|  | Unknown | 9610 | 4.3 | 479 | 3.59 |
| **Ever used hormone replacement therapy (women only)** | Yes | 82466 | 36.9 | 6219 | 46.6 |
|  | No | 140052 | 62.6 | 7069 | 53 |
|  | Unknown | 1177 | 0.53 | 57 | 0.43 |
| **Hypertension** | Yes | 48662 | 21.8 | 3464 | 26 |
|  | No | 174226 | 77.9 | 9838 | 73.7 |
|  | Unknown | 807 | 0.36 | 43 | 0.32 |
| **Dietary intake processed meat** | <=1 per week | 176698 | 79 | 10560 | 79.1 |
|  | >1 per week | 46189 | 20.6 | 2751 | 20.6 |
|  | Unkown | 808 | 0.36 | 34 | 0.25 |
| **Moderate physical activity (minutes/day) ^3^** | mean (SD) | 66.0 (77.6) |  | 69.3 (78.3) |  |
|  | min-max | 0.00-1440 |  | 0.00-1120 |  |
|  | Unknown | 106659 | 25.8 | 7517 | 26.8 |
| **Weekly alcohol intake (grams) ^3^** | mean (SD) | 204 (178) |  | 212 (183) |  |
|  | min-max | 0.00-8206 |  | 0.00-2511 |  |
|  | Unknown | 128205 | 31 | 8049 | 28.7 |

*^1^* *This number includes all cancer sites for the UK Biobank participants and includes a few additional sites to those included in this study*.

*^2^ Based on calculated lifetime cigarette amounts*

*^3^ Missing in these variables were imputed when used as adjustments in models.*

**Table S4**: Descriptive characteristics for systemic inflammation markers, blood cell counts and blood CRP concentrations stratified by age and sex in the UK Biobank, separately for cancer-free participants and cancer cases.

|  |  |  | **Sex** | | **Age groups** | | | | **p-value^1^** |
| --- | --- | --- | --- | --- | --- | --- | --- | --- | --- |
| **Name** | **Total** | **Min-max** | **Total by sex** | | **30-49** | **50-57** | **58-63** | **64+** |  |
|  | **(mean (95% CI))** |  | **(mean (95% CI))** | |  |  |  |  |  |
| **Controls (N)** | |  |  | | **105716** | **109866** | **110386** | **88053** |  |
| SII | 596 (594; 598) | 0.04-212493 | Female | 602 (600; 603) | 651 (649; 654) | 586 (584; 589) | 572 (569; 575) | 600 (597; 604) | <2.2$\times$10^-16^ |
|  |  |  | Male | 589 (585; 592) | 562 (559; 566) | 581 (574; 589) | 599 (590; 608) | 616 (612; 621) | <2.2$\times$10^-16^ |
|  |  |  |  |  |  |  |  |  |  |
| NLR | 2.35 (2.34; 2.36) | 0.00-1101 | Female | 2.25 (2.24; 2.25) | 2.41 (2.40; 2.42) | 2.18 (2.17; 2.18) | 2.15 (2.14; 2.16) | 2.27 (2.26; 2.28) | <2.2$\times$10^-16^ |
|  |  |  | Male | 2.46 (2.44; 2.48) | 2.32 (2.27; 2.36) | 2.39 (2.36; 2.43) | 2.52 (2.48; 2.57) | 2.65 (2.63; 2.66) | <2.2$\times$10^-16^ |
|  |  |  |  |  |  |  |  |  |  |
| PLR | 143 (141; 146) | 1.90-344000 | Female | 145 (142; 148) | 148 (148; 149) | 150 (139; 161) | 140 (140; 141) | 141 (140; 141) | 5.37$\times$10^-02^ |
|  |  |  | Male | 141 (137; 146) | 146 (133; 158) | 142 (133; 151) | 141 (133; 149) | 137 (136; 137) | 6.11$\times$10^-01^ |
|  |  |  |  |  |  |  |  |  |  |
| LMR | 4.99 (4.91; 5.08) | 0.01-5401 | Female | 5.62 (5.49; 5.76) | 5.82 (5.51; 6.13) | 5.65 (5.44; 5.86) | 5.62 (5.35; 5.89) | 5.35 (5.07; 5.63) | 1.40$\times$10^-01^ |
|  |  |  | Male | 4.25 (4.17; 4.34) | 4.54 (4.38; 4.71) | 4.45 (4.25; 4.66) | 4.02 (3.93; 4.12) | 3.96 (3.75; 4.17) | 2.36$\times$10^-07^ |
|  |  |  |  |  |  |  |  |  |  |
| Neutrophils | 4.22 (4.22; 4.22) | 0.00-25.4 | Female | 4.19 (4.19; 4.20) | 4.42 (4.41; 4.43) | 4.08 (4.07; 4.09) | 4.07 (4.06; 4.08) | 4.24 (4.22; 4.25) | <2.2$\times$10^-16^ |
|  |  |  | Male | 4.25 (4.24; 4.26) | 4.07 (4.06; 4.08) | 4.20 (4.18; 4.21) | 4.31 (4.30; 4.32) | 4.45 (4.43; 4.46) | <2.2$\times$10^-16^ |
|  |  |  |  |  |  |  |  |  |  |
| Lymphocytes | 1.96 (1.95; 1.96) | 0.00-85.7 | Female | 2.01 (2.01; 2.01) | 1.95 (1.95; 1.96) | 2.01 (2.01; 2.02) | 2.04 (2.04; 2.05) | 2.03 (2.03; 2.04) | <2.2$\times$10^-16^ |
|  |  |  | Male | 1.89 (1.89; 1.90) | 1.91 (1.91; 1.92) | 1.91 (1.90; 1.92) | 1.88 (1.88; 1.89) | 1.86 (1.86; 1.87) | <2.2$\times$10^-16^ |
|  |  |  |  |  |  |  |  |  |  |
| Monocytes | 0.47 (0.47; 0.47) | 0.00-34.3 | Female | 0.44 (0.44; 0.44) | 0.44 (0.43; 0.44) | 0.43 (0.43; 0.43) | 0.44 (0.44; 0.44) | 0.46 (0.46; 0.46) | <2.2$\times$10^-16^ |
|  |  |  | Male | 0.51 (0.51; 0.52) | 0.48 (0.48; 0.48) | 0.51 (0.50; 0.51) | 0.53 (0.53; 0.53) | 0.55 (0.54; 0.55) | <2.2$\times$10^-16^ |
|  |  |  |  |  |  |  |  |  |  |
| Platelets | 253 (253; 253) | 0.3-1821 | Female | 266 (265; 266) | 269 (268; 269) | 267 (267; 268) | 264 (263; 264) | 262 (261; 262) | <2.2$\times$10^-16^ |
|  |  |  | Male | 238 (237; 238) | 243 (243; 243) | 241 (241; 242) | 236 (235; 236) | 230 (230; 231) | <2.2$\times$10^-16^ |
|  |  |  |  |  |  |  |  |  |  |
| CRP | 2.54 (2.52; 2.55) | 0.08-80.0 | Female | 2.65 (2.63; 2.67) | 2.32 (2.28; 2.35) | 2.60 (2.56; 2.63) | 2.76 (2.73; 2.80) | 2.99 (2.95; 3.04) | <2.2$\times$10^-16^ |
|  |  |  | Male | 2.40 (2.38; 2.42) | 2.12 (2.09; 2.16) | 2.29 (2.25; 2.32) | 2.48 (2.44; 2.52) | 2.76 (2.71; 2.80) | <2.2$\times$10^-16^ |
|  |  |  |  |  |  |  |  |  |  |
| **Cases (N)** | |  |  | | **2609** | **5347** | **9295** | **10843** |  |
| SII | 618 (613; 623) | 1.81-21461 | Female | 614 (607; 621) | 657 (641; 673) | 615 (603; 627) | 590 (577; 603) | 620 (607; 632) | 6.70$\times$10^-08^ |
|  |  |  | Male | 622 (615; 629) | 601 (569; 632) | 608 (592; 625) | 612 (601; 623) | 637 (626; 647) | 1.49$\times$10^-03^ |
|  |  |  |  |  |  |  |  |  |  |
| NLR | 2.44 (2.43; 2.46) | 0.02-92.5 | Female | 2.28 (2.26; 2.31) | 2.41 (2.36; 2.45) | 2.26 (2.22; 2.29) | 2.21 (2.16; 2.26) | 2.33 (2.29; 2.37) | 4.07$\times$10^-07^ |
|  |  |  | Male | 2.58 (2.56; 2.61) | 2.39 (2.30; 2.48) | 2.45 (2.40; 2.50) | 2.53 (2.50; 2.57) | 2.70 (2.66; 2.73) | <2.2$\times$10^-16^ |
|  |  |  |  |  |  |  |  |  |  |
| PLR | 141 (141; 142) | 1.70-5661 | Female | 144 (143; 145) | 149 (146; 152) | 146 (144; 148) | 142 (139; 145) | 142 (140; 145) | 4.80$\times$10^-03^ |
|  |  |  | Male | 139 (138; 140) | 145 (139; 151) | 140 (138; 142) | 138 (137; 140) | 139 (137; 141) | 4.43$\times$10^-02^ |
|  |  |  |  |  |  |  |  |  |  |
| LMR | 5.09 (4.79; 5.39) | 0.02-1901 | Female | 5.68 (5.22; 6.14) | 5.98 (4.21; 7.76) | 6.08 (4.80; 7.36) | 5.50 (4.98; 6.03) | 5.46 (4.80; 6.12) | 7.16$\times$10^-01^ |
|  |  |  | Male | 4.55 (4.15; 4.95) | 4.47 (4.26; 4.68) | 4.24 (4.13; 4.36) | 4.87 (4.00; 5.74) | 4.44 (3.82; 5.05) | <2.2$\times$10^-16^ |
|  |  |  |  |  |  |  |  |  |  |
| Neutrophils | 4.36 (4.34; 4.37) | 0.03-52.0 | Female | 4.27 (4.25; 4.30) | 4.45 (4.38; 4.52) | 4.23 (4.18; 4.28) | 4.17 (4.13; 4.21) | 4.34 (4.29; 4.38) | 7.15$\times$10^-13^ |
|  |  |  | Male | 4.43 (4.40; 4.45) | 4.14 (4.04; 4.24) | 4.34 (4.27; 4.41) | 4.38 (4.34; 4.42) | 4.53 (4.49; 4.57) | 4.73$\times$10^-15^ |
|  |  |  |  |  |  |  |  |  |  |
| Lymphocytes | 2.05 (2.02; 2.07) | 0.03-83.8 | Female | 2.10 (2.07; 2.13) | 1.98 (1.95; 2.01) | 2.05 (2.00; 2.10) | 2.14 (2.08; 2.20) | 2.14 (2.08; 2.21) | 1.72$\times$10^-03^ |
|  |  |  | Male | 2.00 (1.96; 2.04) | 1.92 (1.84; 2.00) | 2.00 (1.93; 2.06) | 2.05 (1.97; 2.12) | 1.98 (1.92; 2.03) | 2.45$\times$10^-01^ |
|  |  |  |  |  |  |  |  |  |  |
| Monocytes | 0.5 (0.49; 0.5) | 0.00-15.6 | Female | 0.45 (0.45; 0.45) | 0.44 (0.43; 0.45) | 0.43 (0.43; 0.44) | 0.45 (0.44; 0.46) | 0.47 (0.46; 0.47) | 1.54$\times$10^-12^ |
|  |  |  | Male | 0.54 (0.53; 0.54) | 0.49 (0.47; 0.50) | 0.52 (0.51; 0.53) | 0.53 (0.52; 0.54) | 0.56 (0.55; 0.57) | <2.2$\times$10^-16^ |
|  |  |  |  |  |  |  |  |  |  |
| Platelets | 251 (250; 252) | 1.1-1402 | Female | 266 (265; 267) | 271 (268; 274) | 270 (267; 272) | 266 (264; 267) | 263 (261; 265) | 8.99$\times$10^-07^ |
|  |  |  | Male | 237 (236; 238) | 246 (241; 250) | 246 (243; 248) | 238 (237; 240) | 233 (231; 234) | 6.14$\times$10^-12^ |
|  |  |  |  |  |  |  |  |  |  |
| CRP | 2.92 (2.86; 2.97) | 0.08-80.0 | Female | 3.04 (2.96; 3.12) | 2.33 (2.13; 2.54) | 3.05 (2.86; 3.24) | 3.02 (2.88; 3.16) | 3.35 (3.20; 3.49) | 3.65$\times$10^-07^ |
|  |  |  | Male | 2.80 (2.73; 2.88) | 2.35 (2.05; 2.64) | 2.52 (2.35; 2.69) | 2.72 (2.59; 2.85) | 3.03 (2.91; 3.16) | 7.15$\times$10^-13^ |

*^1^* *The p-value represents the testing of a linear regression of linear differences in inflammation markers across the age groups.*

*Abbreviations: CI: confidence interval; CRP: C-reactive protein; LMR: lymphocyte-to-monocyte ratio; NLR: neutrophil-to-lymphocyte ratio; PLR: platelet-to-lymphocyte ratio; SII: systemic immune-inflammation index.*

**Table S5**. Estimated hazard ratios (HRs) from crude and adjusted flexible survival models for all systemic inflammatory markers and all cancer cases included in this study from the UK Biobank.

| **Cancer site** | **Ratio** | **Crude models** | | | **Crude + CRP** | | | **Minimally adjusted models ^1^** | | | **Fully adjusted models ^2^** | | |
| --- | --- | --- | --- | --- | --- | --- | --- | --- | --- | --- | --- | --- | --- |
|  |  | **HR (95% CI) ^3^** | **p-value ^4^** | **Cases (N)** | **HR (95% CI) ^3^** | **p-value ^4^** | **Cases (N)** | **HR (95% CI) ^3^** | **p-value ^4^** | **Cases (N)** | **HR (95% CI) ^3^** | **p-value ^4^** | **Cases (N)** |
| Prostate | SII | 1.07 (0.99-1.16) | 6.78×10^-02^ | 4324 | 1.07 (0.99-1.16) | 8.40×10^-02^ | 4132 | 1.07 (0.99-1.16) | 8.57×10^-02^ | 4068 | 1.07 (0.99-1.16) | 8.65×10^-02^ | 4067 |
|  | NLR | 1.07 (0.99-1.15) | 7.73×10^-02^ |  | 1.06 (0.98-1.15) | 1.16×10^-01^ |  | 1.07 (0.98-1.15) | 1.16×10^-01^ |  | 1.06 (0.98-1.15) | 1.18×10^-01^ |  |
|  | PLR | 1.11 (1.03-1.19) | **6.55×10^-03^** |  | 1.11 (1.02-1.19) | **9.56×10^-03^** |  | 1.1 (1.02-1.19) | **1.46×10^-02^** |  | 1.1 (1.02-1.19) | **1.54×10^-02^** |  |
|  | LMR | 0.91 (0.84-0.99) | **2.07×10^-02^** |  | 0.91 (0.84-0.99) | **2.03×10^-02^** |  | 0.91 (0.84-0.99) | **2.08×10^-02^** |  | 0.91 (0.84-0.99) | **2.09×10^-02^** |  |
| Breast | SII | 1.08 (0.99-1.18) | 7.51×10^-02^ | 4237 | 1.06 (0.97-1.17) | 1.94×10^-01^ | 4077 | 1.06 (0.97-1.16) | 2.18×10^-01^ | 4015 | - | - |  |
|  | NLR | 1.05 (0.96-1.15) | 2.92×10^-01^ |  | 1.04 (0.95-1.14) | 3.97×10^-01^ |  | 1.03 (0.94-1.13) | 5.29×10^-01^ |  | - | - |  |
|  | PLR | 1.01 (0.92-1.1) | 8.85×10^-01^ |  | 1 (0.92-1.1) | 9.25×10^-01^ |  | 1.01 (0.92-1.1) | 8.50×10^-01^ |  | - | - |  |
|  | LMR | 0.98 (0.89-1.07) | 5.95×10^-01^ |  | 0.98 (0.89-1.07) | 6.01×10^-01^ |  | 0.98 (0.89-1.07) | 6.43×10^-01^ |  | - | - |  |
| Colorectal | SII | 1.47 (1.34-1.61) | **2.58×10^-16^** | 2401 | 1.33 (1.2-1.48) | **1.37×10^-07^** | 2273 | 1.33 (1.19-1.48) ^5^ | **2.74×10^-07^** | 2225 | 1.32 (1.18-1.47) | **4.82×10^-07^** | 2209 |
|  | NLR | 1.35 (1.22-1.48) | **5.58×10^-10^** |  | 1.26 (1.13-1.4) | **2.12×10^-05^** |  | 1.26 (1.13-1.4) | **2.58×10^-05^** |  | 1.26 (1.13-1.4) | **2.76×10^-05^** |  |
|  | PLR | 1.36 (1.24-1.5) | **9.82×10^-11^** |  | 1.29 (1.17-1.41) | **2.67×10^-07^** |  | 1.28 (1.16-1.41) | **6.83×10^-07^** |  | 1.28 (1.16-1.42) | **5.25×10^-07^** |  |
|  | LMR | 0.75 (0.67-0.83) | **2.12×10^-08^** |  | 0.8 (0.72-0.89) | **3.51×10^-05^** |  | 0.8 (0.72-0.89) | **6.14×10^-05^** |  | 0.81 (0.72-0.9) | **7.73×10^-05^** |  |
| Lung | SII | 1.69 (1.5-1.89) | **4.83×10^-19^** | 1458 | 1.44 (1.26-1.64) | **7.61×10^-08^** | 1401 | 1.38 (1.21-1.57) | **2.13×10^-06^** | 1368 | 1.38 (1.2-1.59) | **5.74×10^-06^** | 1168 |
|  | NLR | 1.53 (1.37-1.7) | **1.90×10^-14^** |  | 1.33 (1.17-1.52) | **1.14×10^-05^** |  | 1.29 (1.14-1.47) | **9.53×10^-05^** |  | 1.32 (1.15-1.51) | **8.64×10^-05^** |  |
|  | PLR | 1 (0.88-1.14) | 9.83×10^-01^ |  | 0.96 (0.84-1.08) | 4.82×10^-01^ |  | 0.93 (0.82-1.05) | 2.33×10^-01^ |  | 1.07 (0.93-1.22) | 3.57×10^-01^ |  |
|  | LMR | 0.87 (0.78-0.97) | **1.43×10^-02^** |  | 0.94 (0.84-1.06) | 3.28×10^-01^ |  | 0.95 (0.85-1.07) | 4.36×10^-01^ |  | 0.92 (0.81-1.04) | 1.85×10^-01^ |  |
| Lymphoma | SII | 0.91 (0.8-1.04) | 1.60×10^-01^ | 910 | 0.85 (0.76-0.97) | **1.21×10^-02^** | 866 | 0.86 (0.76-0.97) | **1.57×10^-02^** | 855 | - | - |  |
|  | NLR | 0.97 (0.86-1.1) | 6.68×10^-01^ |  | 0.94 (0.83-1.06) | 3.04×10^-01^ |  | 0.95 (0.84-1.07) | 3.70×10^-01^ |  | - | - |  |
|  | PLR | 0.94 (0.83-1.06) | 3.09×10^-01^ |  | 0.93 (0.83-1.05) | 2.57×10^-01^ |  | 0.93 (0.82-1.05) | 2.38×10^-01^ |  | - | - |  |
|  | LMR | 0.87 (0.77-1) | **4.37×10^-02^** |  | 0.91 (0.8-1.03) | 1.32×10^-01^ |  | 0.9 (0.8-1.02) | 1.14×10^-01^ |  | - | - |  |
| Endometrial | SII | 1.33 (1.08-1.65) | **8.29×10^-03^** | 650 | 1.16 (0.91-1.47) | 2.27×10^-01^ | 629 | 1.2 (0.94-1.52) | 1.41×10^-01^ | 617 | - | - |  |
|  | NLR | 1.3 (1.04-1.62) | **2.04×10^-02^** |  | 1.13 (0.89-1.43) | 3.20×10^-01^ |  | 1.18 (0.93-1.5) | 1.82×10^-01^ |  | - | - |  |
|  | PLR | 1.21 (0.97-1.52) | 9.61×10^-02^ |  | 1.2 (0.95-1.51) | 1.27×10^-01^ |  | 1.25 (0.99-1.59) | 5.79×10^-02^ |  | - | - |  |
|  | LMR | 0.75 (0.59-0.96) | **2.14×10^-02^** |  | 0.82 (0.63-1.06) | 1.29×10^-01^ |  | 0.79 (0.61-1.03) | 8.26×10^-02^ |  | - | - |  |
| Kidney | SII | 1.61 (1.33-1.93) | **5.31×10^-07^** | 537 | 1.39 (1.11-1.74) | **4.50×10^-03^** | 514 | 1.41 (1.13-1.77) | **2.82×10^-03^** | 504 | 1.39 (1.1-1.75) | **5.10×10^-03^** | 501 |
|  | NLR | 1.45 (1.2-1.75) | **1.15×10^-04^** |  | 1.28 (1.02-1.61) | **3.69×10^-02^** |  | 1.3 (1.03-1.63) | **2.62×10^-02^** |  | 1.29 (1.02-1.62) | **3.20×10^-02^** |  |
|  | PLR | 1.4 (1.18-1.66) | **1.06×10^-04^** |  | 1.31 (1.08-1.59) | **5.37×10^-03^** |  | 1.35 (1.12-1.64) | **1.69×10^-03^** |  | 1.36 (1.12-1.64) | **1.49×10^-03^** |  |
|  | LMR | 0.75 (0.64-0.88) | **4.39×10^-04^** |  | 0.8 (0.67-0.96) | **1.52×10^-02^** |  | 0.79 (0.66-0.94) | **9.90×10^-03^** |  | 0.79 (0.66-0.95) | **1.02×10^-02^** |  |
| Bladder | SII | 1.33 (1.06-1.67) | **1.36×10^-02^** | 497 | 1.21 (0.95-1.55) | 1.23×10^-01^ | 475 | 1.22 (0.95-1.57) | 1.13×10^-01^ | 469 | - | - |  |
|  | NLR | 1.17 (0.93-1.48) | 1.78×10^-01^ |  | 1.09 (0.85-1.39) | 5.00×10^-01^ |  | 1.09 (0.85-1.4) | 4.97×10^-01^ |  | - | - |  |
|  | PLR | 1.11 (0.87-1.41) | 4.09×10^-01^ |  | 1.05 (0.81-1.36) | 7.38×10^-01^ |  | 1.06 (0.82-1.38) | 6.60×10^-01^ |  | - | - |  |
|  | LMR | 0.91 (0.71-1.17) | 4.63×10^-01^ |  | 0.99 (0.77-1.27) | 9.19×10^-01^ |  | 0.98 (0.75-1.26) | 8.54×10^-01^ |  | - | - |  |
| Ovary | SII | 1.44 (1.09-1.91) | **1.11×10^-02^** | 429 | 1.39 (1.04-1.85) | **2.69×10^-02^** | 418 | 1.38 (1.03-1.84) | **3.02×10^-02^** | 413 | 1.33 (0.99-1.78) | 6.00×10^-02^ | 393 |
|  | NLR | 1.45 (1.1-1.9) | **7.90×10^-03^** |  | 1.4 (1.06-1.84) | **1.59×10^-02^** |  | 1.4 (1.06-1.85) | **1.61×10^-02^** |  | 1.34 (1.01-1.77) | **4.32×10^-02^** |  |
|  | PLR | 1.38 (1.05-1.83) | **2.24×10^-02^** |  | 1.41 (1.06-1.86) | **1.73×10^-02^** |  | 1.4 (1.05-1.85) | **1.99×10^-02^** |  | 1.34 (1.01-1.78) | **4.29×10^-02^** |  |
|  | LMR | 0.71 (0.53-0.96) | **2.39×10^-02^** |  | 0.74 (0.55-1.01) | 5.46×10^-02^ |  | 0.74 (0.54-1) | **4.90×10^-02^** |  | 0.72 (0.53-0.98) | **3.60×10^-02^** |  |
| Pancreas | SII | 1.14 (0.87-1.48) | 3.40×10^-01^ | 420 | 0.98 (0.75-1.28) | 9.03×10^-01^ | 405 | 0.97 (0.73-1.27) | 8.10×10^-01^ | 399 | - | - |  |
|  | NLR | 1.12 (0.86-1.45) | 4.18×10^-01^ |  | 1.01 (0.77-1.33) | 9.56×10^-01^ |  | 0.95 (0.71-1.27) | 7.33×10^-01^ |  | - | - |  |
|  | PLR | 1.02 (0.78-1.34) | 8.97×10^-01^ |  | 1.02 (0.77-1.34) | 9.00×10^-01^ |  | 1 (0.75-1.33) | 9.74×10^-01^ |  | - | - |  |
|  | LMR | 0.81 (0.63-1.05) | 1.19×10^-01^ |  | 0.86 (0.66-1.11) | 2.49×10^-01^ |  | 0.91 (0.69-1.2) | 4.87×10^-01^ |  | - | - |  |
| Oesophagal | SII | 1.45 (1.13-1.85) | **2.94×10^-03^** | 393 | 1.32 (1.02-1.71) | **3.27×10^-02^** | 375 | 1.35 (1.05-1.75) | **2.08×10^-02^** | 368 | - | - |  |
|  | NLR | 1.29 (1.01-1.65) | **3.98×10^-02^** |  | 1.21 (0.93-1.55) | 1.51×10^-01^ |  | 1.22 (0.95-1.58) | 1.23×10^-01^ |  | - | - |  |
|  | PLR | 1.22 (0.97-1.54) | 9.60×10^-02^ |  | 1.02 (0.83-1.26) | 8.44×10^-01^ |  | 1.22 (0.97-1.55) | 9.33×10^-02^ |  | - | - |  |
|  | LMR | 0.77 (0.59-1.02) | 6.85×10^-02^ |  | 0.82 (0.62-1.09) | 1.76×10^-01^ |  | 0.81 (0.61-1.08) | 1.55×10^-01^ |  | - | - |  |
| Oral | SII | 0.97 (0.76-1.25) | 8.34×10^-01^ | 381 | 0.93 (0.72-1.21) | 5.92×10^-01^ | 363 | 0.91 (0.7-1.18) | 4.63×10^-01^ | 356 | - | - |  |
|  | NLR | 0.94 (0.72-1.23) | 6.53×10^-01^ |  | 0.91 (0.69-1.2) | 5.10×10^-01^ |  | 0.89 (0.68-1.17) | 3.94×10^-01^ |  | - | - |  |
|  | PLR | 0.81 (0.63-1.04) | 9.80×10^-02^ |  | 0.8 (0.62-1.03) | 8.12×10^-02^ |  | 0.78 (0.61-1) | **4.84×10^-02^** |  | - | - |  |
|  | LMR | 0.97 (0.75-1.25) | 8.02×10^-01^ |  | 0.99 (0.76-1.29) | 9.45×10^-01^ |  | 1 (0.77-1.31) | 9.83×10^-01^ |  | - | - |  |
| Brain | SII | 1.41 (1.06-1.87) | **1.77×10^-02^** | 334 | 1.39 (1.04-1.87) | **2.66×10^-02^** | 320 | 1.44 (1.07-1.94) | **1.74×10^-02^** | 314 | - | - |  |
|  | NLR | 1.4 (1.05-1.86) | **2.06×10^-02^** |  | 1.36 (1.02-1.82) | **3.78×10^-02^** |  | 1.39 (1.03-1.87) | **3.01×10^-02^** |  | - | - |  |
|  | PLR | 1.32 (0.98-1.79) | 6.71×10^-02^ |  | 1.31 (0.97-1.77) | 8.16×10^-02^ |  | 1.34 (0.98-1.83) | 6.69×10^-02^ |  | - | - |  |
|  | LMR | 0.91 (0.67-1.23) | 5.38×10^-01^ |  | 0.92 (0.67-1.25) | 5.96×10^-01^ |  | 0.92 (0.67-1.26) | 6.04×10^-01^ |  | - | - |  |
| Myeloma | SII | 0.82 (0.66-1.02) | 7.13×10^-02^ | 311 | 0.7 (0.59-0.84) | **1.49×10^-04^** | 294 | 0.72 (0.59-0.88) | **1.34×10^-03^** | 289 | - | - |  |
|  | NLR | 0.86 (0.7-1.05) | 1.45×10^-01^ |  | 0.76 (0.62-0.92) | **5.34×10^-03^** |  | 0.78 (0.63-0.97) | **2.40×10^-02^** |  | - | - |  |
|  | PLR | 1.1 (0.83-1.44) | 5.10×10^-01^ |  | 0.89 (0.66-1.2) | 4.59×10^-01^ |  | 0.93 (0.69-1.26) | 6.42×10^-01^ |  | - | - |  |
|  | LMR | 1.06 (0.79-1.43) | 6.76×10^-01^ |  | 0.95 (0.68-1.32) | 7.48×10^-01^ |  | 0.93 (0.67-1.3) | 6.70×10^-01^ |  | - | - |  |
| Stomach | SII | 1.56 (1.17-2.07) | **2.41×10^-03^** | 273 | 1.46 (1.07-2) | **1.72×10^-02^** | 257 | 1.47 (1.07-2.02) | **1.75×10^-02^** | 254 | - | - |  |
|  | NLR | 1.44 (1.09-1.9) | **1.04×10^-02^** |  | 1.37 (1.02-1.85) | **3.87×10^-02^** |  | 1.37 (1.02-1.85) | **3.87×10^-02^** |  | - | - |  |
|  | PLR | 1.13 (0.84-1.51) | 4.30×10^-01^ |  | 1.24 (0.91-1.7) | 1.79×10^-01^ |  | 1.26 (0.92-1.72) | 1.55×10^-01^ |  | - | - |  |
|  | LMR | 0.84 (0.62-1.14) | 2.69×10^-01^ |  | 0.82 (0.59-1.14) | 2.36×10^-01^ |  | 0.82 (0.59-1.13) | 2.25×10^-01^ |  | - | - |  |
| Liver | SII | 0.98 (0.73-1.32) | 8.97×10^-01^ | 202 | 0.85 (0.61-1.17) | 3.08×10^-01^ | 195 | 0.82 (0.58-1.14) | 2.27×10^-01^ | 193 | - | - |  |
|  | NLR | 1.09 (0.78-1.53) | 6.05×10^-01^ |  | 0.89 (0.63-1.27) | 5.30×10^-01^ |  | 0.87 (0.6-1.26) | 4.64×10^-01^ |  | - | - |  |
|  | PLR | 0.82 (0.61-1.11) | 1.99×10^-01^ |  | 0.78 (0.58-1.07) | 1.23×10^-01^ |  | 0.78 (0.57-1.08) | 1.32×10^-01^ |  | - | - |  |
|  | LMR | 0.77 (0.55-1.08) | 1.31×10^-01^ |  | 0.83 (0.6-1.14) | 2.52×10^-01^ |  | 0.81 (0.58-1.13) | 2.17×10^-01^ |  | - | - |  |
| Thyroid | SII | 0.98 (0.67-1.43) | 9.02×10^-01^ | 181 | 0.8 (0.53-1.2) | 2.88×10^-01^ | 172 | 0.81 (0.54-1.22) | 3.20×10^-01^ | 166 | - | - |  |
|  | NLR | 0.91 (0.62-1.34) | 6.44×10^-01^ |  | 0.75 (0.55-1.03) | 7.13×10^-02^ |  | 0.75 (0.54-1.04) | 8.72×10^-02^ |  | - | - |  |
|  | PLR | 1 (0.68-1.47) | 9.94×10^-01^ |  | 0.99 (0.66-1.5) | 9.75×10^-01^ |  | 1.01 (0.67-1.53) | 9.55×10^-01^ |  | - | - |  |
|  | LMR | 1.09 (0.73-1.65) | 6.66×10^-01^ |  | 1.22 (0.79-1.89) | 3.68×10^-01^ |  | 1.21 (0.78-1.88) | 3.89×10^-01^ |  | - | - |  |

*^1^ Adjustment for CRP, educational level and BMI*

*^2^ Site-specific adjustments were performed according Table S2*

*^3^ HR with 95% confidence intervals (CIs) estimated in each model*

*^4^ Significance of the HR, marked with bold text where p<0.05*

*^5^ The estimates for the HRs when excluding extreme values for CRP, regular use of aspirin or lymphocyte counts were 1.32 (1.18-1.48), 1.38 (1.10-1.72) and 1.33 (1.19-1.49), respectively. The sensitivity analyses evaluated influence of those participants with CRP concentrations > 10 mg/L and those with lymphocyte counts less than the first quartile value-1.5*interquartile range or more than the third quartile value+1.5*interquartile range by excluding these participants from the analyses. We excluded 17,026 participants with CRP levels >10 mg/L, and 255 cases were excluded from the minimally adjusted models for colorectal cancer. Correspondingly for extreme lymphocyte counts, we excluded 9,028 participants, and 41 cases were excluded from the minimally adjusted models for colorectal cancer.*

*Abbreviations: BMI: Body mass index; CI: confidence interval; CRP: C-reactive protein; HR: hazard ratio; LMR: lymphocyte-to-monocyte ratio; NLR: neutrophil-to-lymphocyte ratio; PLR: platelet-to-lymphocyte ratio; SII: systemic immune-inflammation index.*
